# Three-dimensional reconstruction of high latitude bamboo coral via X-ray microfocus Computed Tomography

**DOI:** 10.1038/s41597-024-03396-9

**Published:** 2024-06-07

**Authors:** Thomas J. Williams, Philip J. Basford, Orestis L. Katsamenis, Martin Solan, Gavin L. Foster, Christopher Standish, Jasmin A. Godbold, Philippe Archambault

**Affiliations:** 1 School of Ocean and Earth Science, National Oceanography Centre Southampton, University of Southampton, Waterfront Campus, European Way, Southampton, SO14 3ZH UK; 2 µ-VIS X-ray Imaging Centre, Building 5, University of Southampton, Highfield Campus, University Road, Southampton, SO17 1BJ UK; 3 ArcticNet, Québec Océan, Takuvik Joint International Laboratory CNRS, Université Laval, Quebec City, QC Canada

**Keywords:** Marine biology, Ocean sciences

## Abstract

The skeletons of long-lived bamboo coral (Family *Keratoisididae*) are promising archives for deep-water palaeoceanographic reconstructions as they can record environmental variation at sub-decadal resolution in locations where *in-situ* measurements lack temporal coverage. Yet, detailed three dimensional (3D) characterisations of bamboo coral skeletal architecture are not routinely available and non-destructive investigations into microscale variations in calcification are rare. Here, we provide high-resolution micro-focus computed tomography (µCT) data of skeletal density for two species of bamboo coral (*Acanella arbuscula:* 5 specimens, voxel size, 15 µm (central branch scans) and 50 µm (complete structure scan); *Keratoisis* sp.: 4 specimens, voxel size, 15 µm) collected from the Labrador Sea and Baffin Bay deep-water basins, Eastern Canadian Arctic. These data provide reference models useful for developing methods to assess structural integrity and other fine-scale complexities in many biological, geological, and industrial systems. This will be of wider value to those investigating structural composition, arrangement and/or composition of complex architecture within the fields and subdisciplines of biology, ecology, medicine, environmental geology, and structural engineering.

## Background & Summary

Deep-water bamboo corals form complex structures that, as they grow, archive seasonally resolved oceanographic information^1^. This information is important for efforts to reconstruct both recent and ancient environmental conditions^2^. Stands of these corals also play an important role in mediating benthic biodiversity and functioning by enhancing the density of bioturbators and sediment nutrient release^3^. However, a combination of their extended longevity (>100 years^4^) and slow growth rates^5^ mean that populations are vulnerable to physical disturbance^6^ such that intact specimens have seldom been sampled and are not widely available. Yet, detailed information on coral skeletal architecture is vital for understanding calcification strategies and growth patterns^7^ in response to changing environmental circumstance, and can be informative for marine planning and conservation measures^8^.

Techniques used to investigate the microstructure of coral skeletons, such as scanning electron microscopy (SEM) and the grinding of sections, have relied on methods that require high workloads, strict operability and destructive preparation work^9^. Recent imaging methods, such as high-resolution micro-focus computed tomography (µCT) removes these constraints and, as it allows quantitative analyses of coral skeletal microarchitecture, is emerging as a growing area of scientific focus^10,11^ for contemporary investigations of reef-building coral skeletons^12–16^.

This data descriptor presents µCT scans of two species of deep-water bamboo coral (*Acanella arbuscula* and *Keratoisis* sp.) obtained from the Eastern Canadian Arctic. These μCT scans can provide reference models which may be of use in the development of novel structural designs, analysis routines and computer models for fields such as ecology^17^ orthopaedics^18^, environmental geology and structural engineering^19^. The data may also be of particular interest to those investigating radial growth patterns and banding^20^, coral calcification and bioerosion^21^, impacts of climate change on marine calcifiers^22,23^, coral skeletal-canal networks^24^ and coral-to-bone substitute biocompatibility^25^. The data files are provided as a sequence of stacked tagged image file format (TIFF) images for each scan. These tiff stacks can be opened by a variety of software, including Fiji/ImageJ, which includes instructions for opening in the accompanying user manual^26^.

## Methods

**Fig. 1:**
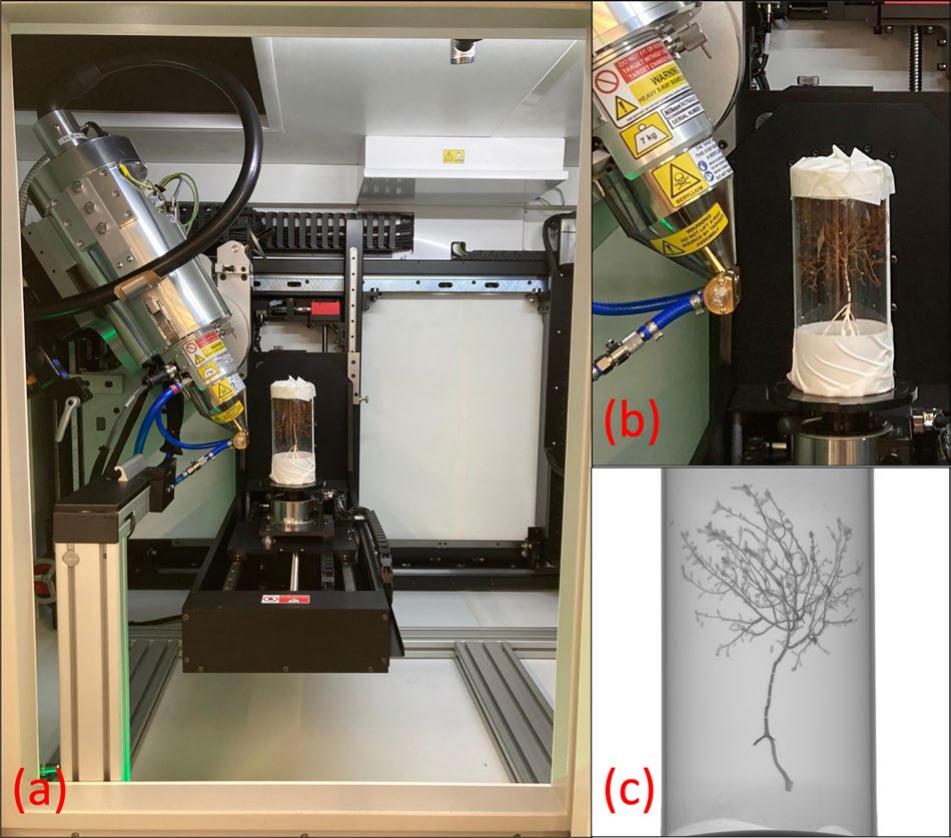
(**a**) Overview, (**b**) Close-up and (**c**) Radiograph of an *Acanella arbuscula* specimen inside a Perspex holding tube in the micro-focus computed tomography scanner housed within the 3D X-ray Histology (XRH) Biomedical Imaging Unit facility at University Hospital Southampton.

Five specimens of *Acanella arbuscula* and four specimens of *Keratoisis* sp. were collected from two deep-water stations (Davis Strait; 63° 20.7198′ N; 58° 11.7426′ W, 1311 m, 3.5 °C, salinity 34.9, 29^th^ July 2021, Disko Fan; 67° 57.9786′ N, 59° 29.6286′ W, 889 m, 1.1 °C, salinity 33.5, 2^nd^ August 2021) using a remotely operated submersible (Sub-Atlantic® Comanche, Forum Energy Technologies^TM^, USA) during the 2021 Amundsen expedition (15^th^ July 2021 – 12^th^ August 2021, *CCGS Amundsen*). These stations reside within the historically heavily fished^27^, and now Marine Conservation Areas (since 2017^28,29^), of the Eastern Canadian Arctic. Permits to Fish for Scientific Purposes were obtained from Fisheries and Oceans Canada (Licence NL-6515-21; Licence S-21/22-1030-NU). *A. arbuscula* is considered an indicator of Vulnerable Marine Ecosystems^30^ whilst *Keratoisis* sp. has not, to date, been found anywhere else in the world^31^. Where possible, the corals were sampled at or close to the basal internode (near the base of the specimen at the sediment surface). Any external debris and residing fauna were carefully removed from the collected colonies using tweezers before each specimen was sealed in a plastic Ziplock bag and frozen at −20 °C. After 72 hours, the specimens were removed from the freezer and carefully cleaned with jets of re-circulated 0.45 µm membrane-filtered seawater (FSW) at 4 °C using a WaterPik^TM^ before being placed back in −20 °C^32^. The cleaned skeleton portions were then sealed in new Ziplock plastic sample bags enclosed in Tupperware (*Acanella arbuscula*) or PVC vinyl tubing (*Keratoisis s*p.) before being transported to the University of Southampton, UK. Here, the specimens were re-housed within Perspex tubes, sealed with polystyrene bungs (Fig. 1), and brought to the μ-VIS X-ray Imaging Centre (www.muvis.org) for µCT scanning. Specifically, imaging took place at the centre’s 3D X-ray Histology (XRH) facility at the University Hospital Southampton^33^, which is a dedicated division for biomedical imaging.

Reconstruction of biogenic structures was achieved using a custom designed Nikon XT micro-focus computed tomography housed within the 3D X-ray Histology (XRH) facility. This system is based on the XT H 225 ST (Nikon Tring, UK). As the system used to acquire the scan data requires the corals to be held vertically, specimens were secured upright in custom-made Perspex holding tubes with polystyrene bungs to ensure stability and prevent movement during rotation (360 degrees) and scanning (Fig. 1). The scans (acquisition time: 15 – 83 minutes; total projections: 2001 – 3501) were all performed at 80 KVp using a Molybdenum target with no filtration. The detector in the scanner is 2850 × 2850 pixels and was used un-binned. For the overview scans of *A. arbuscula* at 50 µm a 12 W power could be used but, for the higher resolution scans (15 µm; *A. arbuscula* and *Keratoisis* sp.) power was reduced to 6.9 W to allow for a sharper (smaller) X-ray focal spot (see Table 1 for more scan parameters). Additionally, a tube of water was scanned at the same time as the samples under the same beam conditions to allow it to be used as a density phantom. The work this data was collected to support focuses on studying the phenotype (microanatomy), which does not require densitometric calibration. However, it was recognised that this may be valuable in the future so the raw data required to calibrate the scans was collected at the same time for futureproofing the datasets. As of now, the data-size limitations set by repositories dictate that access to these raw data files can only be obtained from the authors. All reconstructions were performed using CT Pro 3D 6.6 or 6.7 (Nikon Xtek, Tring UK). The reconstructions were performed using Nikon CT Pro/CT Agent with the beam hardening 4 preset. The software performs a linearisation operation of the beam hardening curves using a pre-determined correction profile. Preset 4 uses the following variables: CoefX4 = 0.0, CoefX3 = 0.0, CoefX2 = 0.8, CoefX1 = 0.2, CoefX0 = 0.0, Scale = 4.44. No additional ring filter or noise filter was specified.

**Table 1:** Typical operating parameters during scans of *Acanella arbuscula* and *Keratoisis* sp. specimens in the custom designed Nikon XT micro-focus computed tomography housed within the 3D X-ray Histology (XRH) facility.

Species	Scan type	Acquisition mode	Isotropic voxel edge size (µm)	Isotropic voxel edge size (µm)	Beam Energy (KVp)	X-ray Power (W)	Number of projections	Frames per projection	Exposure time per frame (ms)	Approx. total time per acquisition (min)
Acanella arbuscula	Complete structure	Circular (360°) CT	50	50	80	12	2501	4	89	15
	Central branch	Circular (360°) CT	15	15	80	8.9	2001	4	125	17
Keratoisis sp.	Complete structure	Circular (360°) CT	15	15	80	8.9	3501	4	354	83

The field of view for the desired resolution did not allow the full height of the *Keratoisis* sp. samples (11.1 – 24.5 cm) to be scanned in a single scan, so multiple overlapping vertical scan positions (n = 3) were used which were then concatenated after reconstruction. The chosen overlap was designed specifically to exclude cone-beam under-sampling artifacts that occur at the top and bottom of the reconstructed space from the concatenated volume. The concatenation was performed using a custom written macro for Fiji titled ‘AutomaticConcatenationPlusIntensityEqualisation’ from the XRH toolbox^34^, which enables the user to manually or automatically select the fusion slice on each volume. If textural information is sufficient and variation from slice to slice significant, the selection can be done automatically. If not, the user can select to bypass the automatic slice selection and select the fusion slice manually. The script then crops the bottom volume between “slice one” and up to the “selected slice”, and top volume from “selected slice” up to “last slice”, and before stitching them into a single volume adjusts the contrast and brightness of the first image of the top volume to match that of the last image of the bottom volume. This ensures a smooth transition from one volume to the other and corrects the intensity variations caused by the heel effect. Intensity calibration is carried out by sampling regions of interest (ROIs) and fitting a straight line using mean intensity values. The parameters obtained from the calibration are applied to the “top” stack to linearly shift the intensity window of the top volume. The two stacks are subsequently concatenated into a single stack and a preview of the concatenated stack is generated by performing a radial reslice to allow the user to evaluate the “smoothness” of the transition. The process can then be repeated to concatenate a third volume onto of the resulted volume-1 + volume-2 volume, etc. Following concatenation on the 32-bit, the resulted volume it was converted to 8-bit in Fiji/ImageJ (v 1.53c^26^) to reduce the data size making it easier to process. These complete volumes were then exported as tiff stacks to enable upload into the Polar Data Centre^35^, as such, the macro does not need to be run a second time on the data files.

**Fig. 2:**
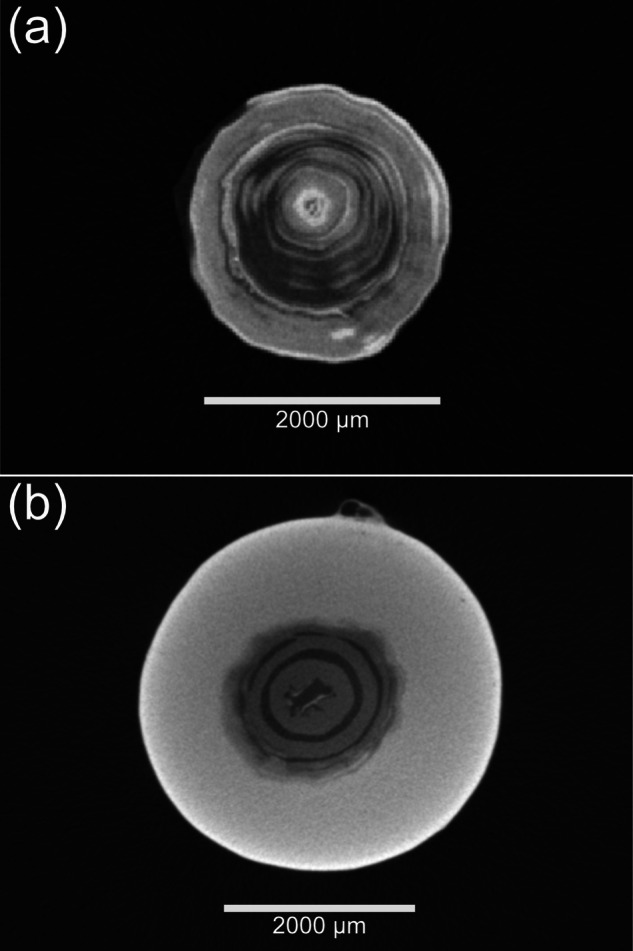
A scaled transverse slice (voxel size, 15 µm) from the (**a**) *Acanella arbuscula* 8-bit coral volumes image set and (**b**) *Keratoisis* sp. 8-bit coral volumes image set showing rings of low density organic tissue (dark grey) and higher density calcium carbonate (light grey) at the node-internode connection, viewed in Fiji^26^ (v2.3.0). Each coral volume image set consists of numbered images that are sequentially stacked to create the three-dimensional coral model.

**Fig. 3:**
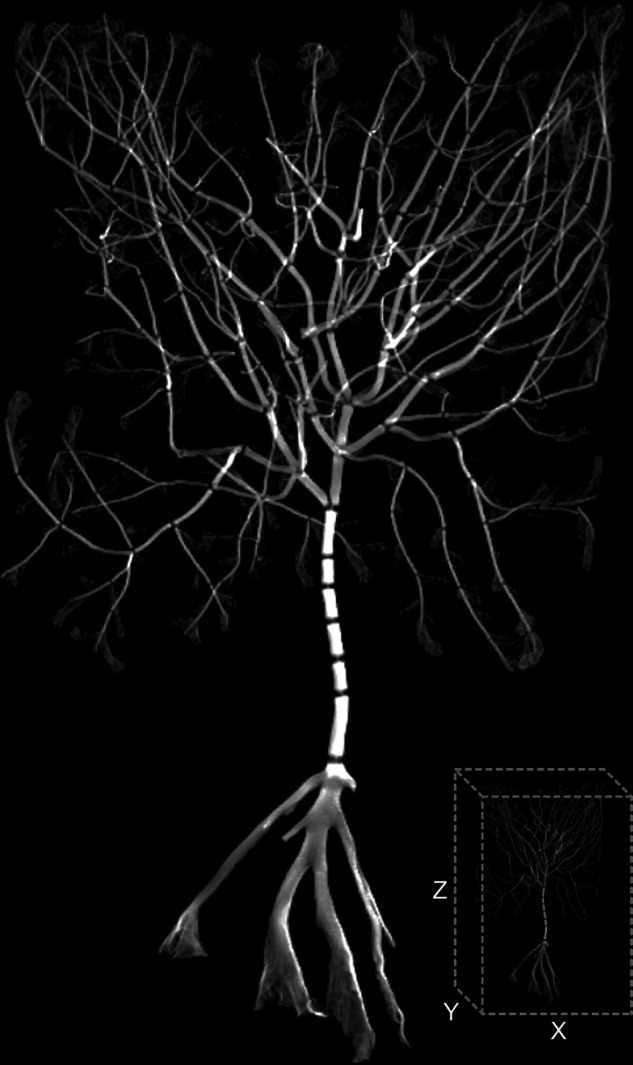
Representative example of reconstructed three-dimensional coral model for *Acanella arbuscula* created from the stacked 8-bit coral volumes images in Dragonfly (v2022.1); approximate dimensions 89 × 85 × 142 (XYZ) mm.

In the stacked images (Fig. 2) and three-dimensional volumes (Fig. 3), levels of grey scale reflect the level of X-ray attenuation caused by variation in bulk density. In this case, brighter pixels represent denser material (calcium carbonate) with darker pixels representing less dense material (organic tissue). To refine coral visualisations, the three-dimensional image captured of the holding tube can be discarded during image processing to leave the skeletal volume (Fig. 3).

## Data Records

All data records (in addition to information regarding data structure, file names, and folder structure) listed in this section are available at the Polar Data Centre^35^. To override the default maximum number of displayed files (n = 1000) in each sub-directory, add the following string “&max = N” to the end of the repository URL, where “N” is the number of files you would like to access. Computed tomography three-dimensional 8-bit volumes have been converted to stacked tagged image file format (TIFF) images with associated dimension data (image width, image breadth, stack height) and scan information presented in portable document format reports (pdfs) to enable access by multiple processing programs. There are five sets of images for *A. arbuscula* complete structure (n = 5), five sets of images for *A. arbuscula* central branch (n = 5) and 4 sets of images for *Keratoisis* sp. (n = 4).

## Technical Validation

### µ-CT calibration

Regular quality assurance inspections are carried out on the µ-CT scanner to verify its metrological and geometrical (alignments) accuracy for conducting the scans. The geometry of source to object and source to detector distances are verified whenever there is any significant physical interaction with the source such as re-alignment, change of filament, or source anode change. This calibration process involves scanning a specially designed phantom known as an ‘hourglass’^36^, which consists of three pairs of high-sphericity spheres. The sphere sizes are as follows: two spheres with a diameter of 3.000 mm, two spheres with a diameter of 6.000 mm, and two spheres with a diameter of 9.525 mm, and each sphere is kept in contact with its size-counterpart. By using this phantom, it becomes possible to accurately determine a known distance, specifically the centre-to-centre distance of the spheres, in a threshold-independent manner. If the measured distance deviates beyond the acceptable limits of metrological accuracy, the system’s calibration parameters are adjusted to ensure agreement between the measured distance and the actual distance.

## Usage Notes

The software options suitable for analysing the data files range from open-source suites, such as Fiji/ImageJ^26^, ITK Snap^37^ or HOROS® (The Horos Project) to commercial software suites such as VGSTUDIO MAX (Volume Graphics), Avizo® (Thermo Fisher Scientific), Simpleware (Synopsys Inc), OsyriX® (Pixmeo), or Dragonfly (Object Research Systems). For instructions on how to open the files please refer to the user manual of the software chosen. The toolbox containing the custom written macro “AutomaticConcatenationPlusIntensityEqualisation” has a file which summarises the functionality of each script and gives an overview of the options available for each script^34^.

## Data Availability

The code used for the concatenation of scans is available as part of the XRH toolbox at 10.5281/zenodo.11148752^34^. **Concatenation code description** A high-level overview of the custom concatenation code is given below. This can be used as template to reproduce the code in any language the reader is more familiar with. **Start** 1. Prompt user to select the “BOTTOM” stack and store its title and bit depth. 2. Prompt user to select the “TOP” stack and store its title and bit depth. 3. Set measurements for analysis. 4. Create a dialog box to configure options. 5. Retrieve selected options from the dialog box. 6. If bit depths are different, display error message and exit. 7. If manual XY translation option is selected: a. Set the measurement tool to a point. b. Prompt the user to select a point of alignment in the “btm” stack and measure its coordinates. c. Prompt the user to select a point of alignment in the “top” stack and measure its coordinates. d. Calculate the translation values and convert them to pixel units. e. Translate the “top” stack using the calculated translation values. 8. If ***automatic slice*** selection option is selected: a. Prompt user to navigate to fusion point in “btm” stack. b. Create reference image from selected slice. c. Normalize reference image. d. Normalize each slice in “top” stack. e. Subtract reference image from “top” stack. f. Calculate standard deviation for each slice. g. Find slice with minimum standard deviation. 9. If ***manual slice selection*** option is selected: a. Prompt user to navigate to fusion point on both “top” and “bottom” volumes and retrieve the slice numbers. 10. Create duplicates of “btm” and “top” stacks by cropping btm volume between “slice 1” up to the “selected slice”, and “top” volume from “selected slice” up to “last slice” 11. Perform intensity calibration by sampling ROIs and fitting a straight line. 12. Apply intensity calibration parameters to “top” stack. 13. Concatenate cropped “btm” and cropped & calibrated “top” stacks into single stack. 14. Perform preview concatenation by creating radial reslice. **End**
